# Supplementary material for: Predicting antifouling paint particle contamination based on 16S rRNA gene sequencing data using random forest-based machine learning
Source: Microbiol Spectr. 2026 Aug 3;14(9):e01578-26. doi: 10.1128/spectrum.01578-26 (PMC13532336; doi:10.1128/spectrum.01578-26)
Supplement: Supplementary file 1 [file spectrum.01578-26-s0001.docx]

**Predicting Antifouling Paint Particle Contamination based on 16S rRNA Sequencing Data using Random Forest-Based Machine Learning**

Sperlea, T.^1^, Labrenz, M^1,2^, Kreikemeyer, B.^3^, Schenk, A.^1^ and Tagg, A. S.^1^*

^1^ Leibniz Institute for Baltic Sea Research Warnemünde, Rostock 18119, Germany

^2^ Marine Research Institute, Klaipėda University, Universiteto al. 17, 92294 Klaipėda, Lithuania

^3^ Institute of Medical Microbiology, Virology and Hygiene, University of Rostock, 18057 Rostock, Germany

*Corresponding author: dralexandertagg@gmail.com

**Supplementary Information**

**Table S1.** Indicator analysis results between combinations of sample types. Index refers to the numeric ID for different combinations of sample group comparisons. Index 14 refers to APP-presence indicators (i.e. low, medium and high vs control). Kingdom column removed since every taxonomy in the table belongs to Bacteria. Statistic refers to the association/fidelity statistic (higher is better) and p.value refers to the P value of the permutation test (p-value significance cut-off 0.05; 999 permutations).

Table S1 continued

Table S1 continued

Table S1 continued

**Table S2.** NCBI BLAST best hits for all *Lutibacter* ASVs, organised by APP indicator status.
**Table S3**: A: Model prediction for microcosm data using binary (presence vs absence) data classification (presence = TRUE; absence = FALSE); B: Model prediction for sampling campaign data using binary (presence vs absence) data classification (APP presence = TRUE; APP absence = FALSE).

**A	B**
